# Vitreous hemorrhage in a pregnant woman with a history of simultaneous pancreas and kidney transplantation: A case report

**DOI:** 10.1016/j.crwh.2022.e00474

**Published:** 2022-12-14

**Authors:** Wajd Aljabri, Saeed Baradwan, Alyaa Alkaff

**Affiliations:** Department of Obstetrics and Gynecology, King Faisal Specialist Hospital and Research Centre, Jeddah, Saudi Arabia

**Keywords:** Pancreas and kidney transplantation, Vitreous hemorrhage, Pregnancy, Multidisciplinary, SPKT, Simultaneous pancreas and kidney transplantation, ESRD, End-stage renal disease, CS, Cesarean section, HELLP, Hemolysis, Elevated Liver enzymes and Low Platelets

## Abstract

Pregnancy after simultaneous pancreas and kidney transplantation (SPKT) carries a high risk of maternal and fetal complications. We report the case of a 39-year-old woman with three consecutive pregnancies with favorable outcomes after SPKT. Within the first year of SPKT, the patient had a spontaneous pregnancy. At 32 weeks of gestation, she underwent an emergency cesarean section (CS) due to severe preeclampsia and HELLP syndrome. The infant was of average birth weight and was transferred to the neonatal intensive care unit for further management. A second unplanned pregnancy occurred almost nine months after the first. The antenatal assessments for fetal growth, blood glucose, and blood pressure were normal throughout follow-up. Early in her pregnancy, the patient developed an uneventful retinopathy of the left eye. At 37 weeks of gestation, she underwent an elective CS due to a short inter-pregnancy interval and delivered a healthy baby with an average birth weight. At the age of 39 years, the patient had a third unplanned pregnancy. She was diagnosed with seronegative antiphospholipid syndrome. She suffered from bilateral vitreous hemorrhage and was managed successfully with a minimally invasive laser treatment combined with an intravitreal injection of anti-vascular endothelial growth factor during her third trimester. At 35 weeks of gestation, the patient presented with labor pain and underwent an emergency CS and delivered a healthy baby with an average birth weight. Pregnancy after SPKT requires a multidisciplinary approach with a careful workup.

## Highlights

• Pregnancy after simultaneous pancreas and kidney transplantation (SPKT) is possible.
• Pregnancy after SPKT carries a high risk of complications for both the mother and the fetus.
• The highest risk of adverse pregnancy outcomes is within the first year after SPKT.
• Management of pregnant women with SPKT requires a multidisciplinary approach.

## 1 Introduction

Simultaneous pancreas and kidney transplantation (SPKT) is usually offered to patients with type-1 diabetes mellitus or end-stage renal disease (ESRD) [1]. Menstrual irregularity is a common feature among women with SPKT [2]. Conception and uneventful pregnancy are often perceived to be difficult after SPKT [1].

Pregnancy after SPKT carries a high risk of complications for both the mother and the fetus. Maternal complications comprise hypertension, infection, preeclampsia, and preterm delivery, while fetal complications comprise low birth weight and early childhood problems [2]. The potential for complications means that antenatal care must be scrupulous [2], with close follow-up during pregnancy, typically by a multidisciplinary team that consists of transplant specialists, nephrologists, endocrinologists, obstetricians, and perinatologists [3].

The first case report of pregnancy following SPKT was published in 1986 [2]. Successful pregnancies and good maternal-fetal outcomes despite the concurrent polypharmacy and immunosuppressive therapy have been reported since that first case [4]. A second pregnancy after SPKT is rare and has not been frequently described [5]. We report the case of a patient who had three successful pregnancies with favorable outcomes after a SPKT.

## 2 Case Presentation

A 39-year-old Middle Eastern woman had been diagnosed with type-1 diabetes mellitus 20 years previously. The course of her disease had deteriorated, complicated by nephropathy and hypertension. She had then developed end-stage renal disease (ESRD) and received hemodialysis for two years. At the age of 34, she underwent a SPKT from a cadaveric donor and thereafter was followed up. After thorough physical and lab investigations, her immunosuppressive medications were adjusted to mycophenolate mofetil 1000 mg once daily, prednisolone 15 mg once daily, tacrolimus 2 mg once daily, and azathioprine 50 mg once daily. She was also prescribed aspirin 81 mg once daily, clopidogrel 75 mg once daily, omeprazole 20 mg once daily, and calcium carbonate 600 mg twice daily. Before the patient's SPKT, she had experienced six consecutive first-trimester abortions with no further complications, and no medical or surgical interventions had been needed. She also had a history of chronic hepatitis B for six years that was treated with entecavir 0.5 once daily.

Within the first year of the SPKT, the patient had a spontaneous, unexpected pregnancy. She presented late for her antenatal follow-up, and was diagnosed with preeclampsia. Her immunosuppressive medications included prednisolone 10 mg once daily, tacrolimus 2 mg once daily, and azathioprine 50 mg once daily. Other medications such as aspirin, clopidogrel, omeprazole, and calcium carbonate were continued with no further dose adjustments. There was average fetal growth and reasonable glycemic control throughout the pregnancy. However, the patient complained of sudden weight gain, bilateral edema of the extremities, shortness of breath, and headache in her third trimester. Her high blood pressure was treated acutely with both labetalol 200 mg once daily and methyldopa 250 mg thrice daily. A renal profile test was abnormal. She completed a dexamethasone course for fetal lung maturity before she underwent an emergency cesarean section at 32 weeks of gestation due to severe preeclampsia and HELLP (Hemolysis, Elevated Liver enzymes and Low Platelets) syndrome. A preterm baby boy (birth weight = 1642 g, 26th percentile) was delivered and then transferred to the neonatal intensive care unit (NICU). During her postpartum hospital stay, the patient had hyperkalemia that was treated immediately. After discharge, her kidney function, blood pressure, and other lab investigations improved in a matter of days. The baby was discharged after three weeks in a good condition and was successfully breastfed.

A second unplanned pregnancy occurred almost nine months after the first. The patient continued on the same dosages of all her immunosuppressive drugs and other prescribed medications, except that the aspirin dose was changed to 100 mg once daily. Her antenatal assessments for fetal growth, blood glucose levels, and blood pressure were normal throughout follow-up. There was no sign of abnormal kidney function or an unstable pancreatic graft function. Early in her pregnancy, she developed retinopathy of the left eye with small vitreous hemorrhage and proliferative changes; no treatment was necessary. At 37 weeks of gestation, the patient underwent an elective cesarean section due to a short inter-pregnancy interval and delivered a healthy baby girl (birth weight = 2921 g, 66th percentile). Her postpartum period was uneventful, and all lab results were within the normal range.

At the age of 39 years, she had a third unplanned pregnancy. She was diagnosed with seronegative antiphospholipid syndrome. Enoxaparin 40 mg twice daily was added to her medications during her pregnancy. In the third trimester, she was maintained on aspirin 100 mg once daily and omeprazole 40 mg once daily. Prednisolone was adjusted to 5 mg once daily at 28 weeks of gestation. The patient suffered from bilateral vitreous hemorrhage and was managed with a minimally invasive laser treatment combined with an intravitreal injection of anti-vascular endothelial growth factor (anti-VEGF) during the last trimester of her pregnancy. The enoxaparin dose was adjusted to once daily as a precaution to avoid any recurrent vitreous hemorrhage. Fetal growth and glycemic control remained normal throughout the pregnancy. At 35 weeks of gestation, the patient presented with labor pain. She subsequently underwent an emergency cesarean section and delivered a healthy baby girl (birth weight = 2687 g, 84th percentile). While the patient's kidney function remained stable, her creatinine levels started to rise after delivery, but were normalized upon discharge, and all other investigations were reassuring. During follow-up, her symptoms improved and she was able to perform her normal daily activities.

## 3 Discussion and Conclusions

Females with ESRD due to diabetic nephropathy usually suffer from reduced fertility [1]. The underlying cause is attributed to the disturbance of the hormonal and metabolic states. After SPKT, the normoglycemic state may be reached, allowing fertility to be drastically improved. Due to the complicated nature of these cases, patients should be closely monitored by a multidisciplinary team before conception and throughout pregnancy [1]. We have reported the case of a woman who had three successful consecutive pregnancies after SPKT. All three had good maternal-fetal outcomes while ensuring graft safety. However, the first pregnancy was complicated by HELLP syndrome and preeclampsia, and resulted in preterm delivery.

Preeclampsia has been reported to be common after SPKT, with a rate of 34% [2]. However, the patient in the present case had two successive pregnancies with standard blood pressure control. The last pregnancy was complicated by vitreous hemorrhage but that was managed successfully with a minimally invasive laser treatment combined with an intravitreal injection of anti-VEGF. Later, the dose of enoxaparin was adjusted to once daily as a precaution to avoid any recurrent vitreous hemorrhage. Preconception planning is considered mandatory in these high-risk cases [5].

In the present case, all three pregnancies were unexpected, and so there was no prior counseling or appropriate investigations. As previously reported, the optimal time to commence any pregnancy in a post-kidney transplant patient is two years after transplantation [5]. Furthermore, the highest risks of graft loss and adverse pregnancy outcomes are within the first post-transplant year [3]. Nevertheless, in the present case, the first pregnancy was within the first year of graft transplant, and there were short intervals between the other two pregnancies. There are insufficient data regarding the long-term effects of short pregnancy intervals on graft outcomes [5]. Both graft function and pregnancy outcomes were favorable in all three pregnancies in the present case.

Some immunosuppressive medications carry an increased risk of teratogenicity. For example, mycophenolate mofetil may cause structural malformations such as shortened fifth fingers, cleft palate, and ear deformities [2]. Therefore, a multidisciplinary meeting should be scheduled before conception to discuss therapy and doses, determine the patient's renal function baseline, and balance the risk of graft rejection and the teratogenic risk of immunosuppressive medications. In the present case, all the medications that the patient took during her pregnancies had no evidence of teratogenicity, and their benefits outweighed the risks. All three fetuses had normal morphology and largely normal growth parameters. Therefore, an expected vaginal delivery should be the goal for all SPKT recipients, although the majority of deliveries are by cesarean section. Most patients have standard glycemic control, although patients using both tacrolimus and prednisolone are reported to have gestational diabetes mellitus more than others [3]. In the present case, the patient was using both medications and maintained a normoglycemic state throughout pregnancy and postpartum.

In conclusion, we have reported the unique case of a patient who had three successful pregnancies with favorable outcomes after a SPKT. However, a multidisciplinary approach is recommended with a careful workup due to the high risks to the mother and fetus.

## Contributors

Wajd Aljabri contributed to the literature review, data acquisition, and drafting of the manuscript.

Saeed Baradwan contributed to the conception of the case report, the literature review, data acquisition, and drafting of the manuscript.

Alyaa Alkaff was involved in patient care, interpreted the data, and revised the article critically for important intellectual content.

All authors approved the final submitted manuscript.

## Funding

This work did not receive any specific grant from funding agencies in the public, commercial, or not-for-profit sectors.

## Patient consent

Written informed consent was obtained from the patient for the publication of this case report.

## Provenance and peer review

This article was not commissioned and was peer reviewed.

## Conflict of interest statement

The authors declare that they have no conflict of interest regarding the publication of this case report.
